# Teledermoscopy-Assisted Referral for Cutaneous Melanoma: Diagnostic Timeliness, Histopathologic Severity, and Stage at Excision in a Comparative Cohort

**DOI:** 10.3390/jcm15103970

**Published:** 2026-05-21

**Authors:** Roxana Grigore, Alexandra Laura Mederle, Roxana Manuela Fericean, Adrian Cosmin Ilie, Emil Florin Hut, Mihail-Alexandru Badea

**Affiliations:** 1 Doctoral School, “Victor Babes” University of Medicine and Pharmacy, Eftimie Murgu Square 2, 300041 Timisoara, Romania; roxana.grigore@umft.ro; 2 Discipline of Dermatology, Center for the Morphologic Study of the Skin (MORPHODERM), Faculty of Medicine, “Victor Babes” University of Medicine and Pharmacy, Eftimie Murgu Square 2, 300041 Timisoara, Romania; alexandra.mederle@umft.ro; 3 Department III Functional Sciences, Division of Public Health and Management, Faculty of Medicine, “Victor Babes” University of Medicine and Pharmacy, 300041 Timisoara, Romania; ilie.adrian@umft.ro; 4 Department of Surgery I, “Victor Babes” University of Medicine and Pharmacy, Eftimie Murgu Square 2, 300041 Timisoara, Romania; 5 Dermatology Department, The George Emil Palade University of Medicine, Pharmacy, Science, and Technology, 540139 Targu Mures, Romania; mihail.badea@umfst.ro

**Keywords:** melanoma, telemedicine, dermoscopy, diagnosis, early, referral and consultation

## Abstract

**Background/Objectives**: Teledermoscopy may improve melanoma triage by accelerating specialist review and compressing the time to definitive treatment, but its clinical relevance depends on whether faster access is accompanied by detection at a less advanced stage. This study compared teledermoscopy-assisted and conventional referral pathways for cutaneous melanoma as a pathway-level service evaluation. **Methods**: In this single-center observational cohort, 87 patients with histologically confirmed primary cutaneous melanoma were analyzed, including 43 managed through teledermoscopy-assisted referral and 44 through a conventional pathway. Primary outcomes were time from referral to dermatology consultation, biopsy, and definitive excision. Secondary outcomes included Breslow thickness, mitotic rate, ulceration, stage distribution, early-stage disease, and selected pathway-quality indicators. **Results**: Teledermoscopy-assisted referral was associated with shorter median times to consultation (9.0 vs. 18.7 days), biopsy (16.6 vs. 30.3 days), and excision (26.9 vs. 43.2 days), all *p* < 0.001. Patients in the teledermoscopy group had lower Breslow thickness (0.7 vs. 1.5 mm, *p* < 0.001), lower mitotic rate (1.2 vs. 2.9 mitoses/mm^2^, *p* < 0.001), a higher proportion of stage 0/I melanoma (79.1% vs. 40.9%; risk ratio 1.93, 95% CI 1.31–2.85), and fewer lesions with Breslow > 2.0 mm (9.3% vs. 36.4%; risk ratio 0.26, 95% CI 0.09–0.70). **Conclusions**: In this non-randomized cohort, teledermoscopy-assisted referral was associated with faster melanoma care and a more favorable stage profile at excision. Because pathway assignment was not randomized and lesion-level referral urgency was incompletely measured, these findings should be interpreted as associations that support further prospective evaluation rather than as proof of causal stage migration.

## 1. Introduction

Melanoma remains one of the most time-sensitive malignancies encountered in dermatologic practice because prognosis is strongly linked to disease burden at diagnosis. Even modest increases in Breslow thickness can translate into higher nodal risk, more extensive staging requirements, and greater treatment intensity. Contemporary staging frameworks and global epidemiologic data consistently reinforce that tumor thickness, ulceration, and stage at presentation remain central determinants of prognosis and service planning in cutaneous melanoma [1,2,3].

In routine care, however, the pathway from first suspicion to definitive excision is often fragmented. Patients may initially present to primary care, urgent care, emergency settings, or non-dermatology specialists, and every handoff introduces opportunities for delay. Time-based cancer referral standards were introduced precisely because earlier recognition, faster specialist review, and timely treatment are clinically meaningful quality targets rather than merely administrative metrics. Across oncology more broadly, treatment delay is associated with worse outcomes, and melanoma-specific cohort data similarly suggest that prolonged surgical intervals may adversely affect prognosis in selected settings [4,5,6,7,8].

Teledermatology has emerged as a practical response to these access barriers, particularly in systems with limited specialist availability or wide geographic dispersion of patients. Among its several forms, teledermoscopy is especially attractive for pigmented lesions because it combines clinical photography with dermoscopic imaging, allowing a dermatologist to assess lesion architecture before the patient is physically seen. Systematic reviews show that image-enabled dermatology pathways can achieve clinically useful diagnostic agreement and triage performance for skin cancer, especially when dermoscopy and standardized workflows are integrated into service design [9,10,11,12,13].

The clinical relevance of teledermoscopy depends not only on whether it shortens access to consultation but on whether it compresses the broader diagnostic arc. For melanoma, the most meaningful operational milestones include time from referral to dermatology consultation, time to biopsy, and time to definitive excision. A pathway can appear efficient if patients reach a dermatologist quickly, yet still fail to improve clinically important outcomes if biopsy, pathology, or surgical management is delayed. Teledermoscopy is therefore best judged as a pathway intervention, not simply as a communication tool, and current consensus guidance emphasizes the importance of standardized workflows, triage thresholds, documentation, and safety-netting when such systems are implemented at scale [10,11,12,13,14].

Pathway redesign is also relevant from an access and equity perspective. Patients living farther from specialist centers or navigating disrupted health systems are more likely to experience delays between suspicion, specialist review, and definitive treatment. Teledermatology has been proposed as one way to reduce these penalties by enabling earlier expert input without requiring immediate in-person capacity, although its benefits depend on image quality, digital access, and equitable workflow implementation. These considerations have become even more visible in recent years, when teledermatology was discussed both as a mitigation strategy and as a domain in which disparities must be actively monitored [12,14,15].

The present manuscript was designed as a melanoma pathway-level service evaluation rather than a randomized efficacy trial. The aim was to compare a teledermoscopy-assisted referral pathway with a conventional referral pathway in a cohort of 87 patients with histologically confirmed cutaneous melanoma. We hypothesized that teledermoscopy-assisted referral would be associated with shorter intervals to consultation, biopsy, and definitive excision. We also explored whether the pathway was associated with differences in histopathologic severity and stage at excision, while recognizing that non-randomized pathway allocation limits causal inference regarding stage migration.

## 2. Materials and Methods

### 2.1. Study Design and Setting

This manuscript presents an observational comparative cohort study performed within a single-center dermatology and dermato-oncology service evaluation. The study compared two referral strategies used for patients with clinically suspicious pigmented lesions who were ultimately diagnosed with primary cutaneous melanoma. The teledermoscopy-assisted pathway consisted of referral with standardized clinical and dermoscopic images submitted for asynchronous dermatologist review before in-person consultation. The conventional pathway consisted of the usual written referral process without pre-consultation image triage. The final analytic cohort included 87 patients with complete core timing and histopathology data: 43 managed through the teledermoscopy-assisted pathway and 44 managed through the conventional pathway.

### 2.2. Participants, Eligibility, and Pathway Workflow

Adults with newly diagnosed primary cutaneous melanoma were eligible when the medical record contained the referral date, dermatology consultation date, biopsy date, definitive excision date, and core histopathologic variables. Patients were excluded if they had recurrent melanoma, mucosal melanoma, ocular melanoma, metastatic melanoma of unknown primary, or major missing timing or pathology data. In the teledermoscopy-assisted pathway, macroscopic clinical photographs and polarized dermoscopic images were obtained by trained referring clinicians or dermatology staff using a standardized image set. The image set included at least one regional/localization image, one close-up clinical image, and one focused dermoscopic image of the index lesion. Images were considered adequate when the lesion was centered, in focus, sufficiently illuminated, and accompanied by patient age, sex, lesion location, clinical history, and the referrer’s level of concern. Image-assisted referrals were reviewed asynchronously by a dermatologist with dermoscopy experience. Triage priority was based on lesion morphology and clinical risk features, including asymmetry, atypical network or structureless areas, irregular dots/globules, blue-white veil, regression structures, atypical vascular pattern, rapid change, symptoms, and high clinical suspicion stated by the referrer. Cases judged highly suspicious were prioritized for earlier in-person assessment and biopsy planning. The conventional pathway was defined as referral based on usual written clinical information, with urgency determined through the standard referral process without image-assisted pre-consultation review. Both pathways required subsequent in-person assessment and histopathologic confirmation. Images were acquired in routine practice using dermatoscope–camera combinations available in the service, typically smartphone-coupled dermoscopy or digital camera-based dermoscopic capture, while preserving the requirement for both clinical overview photography and polarized dermoscopic visualization of the index lesion. Histopathology was reviewed within the institutional pathology service by a restricted pathology/dermatopathology team using the department’s structured melanoma reporting approach, which routinely documents Breslow thickness, ulceration, mitotic activity, and other stage-relevant features used in this analysis.

The workflow was analyzed as a sequence of directly observed dates rather than as an assumed process. Referral-to-consultation, referral-to-biopsy, and referral-to-definitive excision were the main pathway intervals. Consultation-to-biopsy and biopsy-to-excision were also analyzed to determine whether any pathway acceleration was restricted to initial triage or persisted after the first specialist contact. Baseline variables were used descriptively to characterize case mix; the groups were not individually matched, and the design could not fully capture lesion conspicuity, referrer suspicion, patient delay before referral, or calendar-time changes in service capacity.

### 2.3. Variables and Outcome Measures

Primary outcomes were the time intervals, measured in days, from referral to dermatology consultation, from referral to biopsy, and from referral to definitive excision. The referral date was defined as the date on which the referral request entered the institutional referral/scheduling system; for image-assisted referrals, this was the date on which the complete teledermoscopy referral package was received. Dermatology consultation was defined as the first documented in-person dermatologist assessment of the index lesion and did not include the asynchronous image review itself. Biopsy was defined as the date of the diagnostic biopsy or diagnostic excision that generated the histopathologic melanoma diagnosis. Definitive excision was defined as the date of wide local excision or definitive surgical excision intended to complete local management according to pathology and surgical planning. These definitions were applied identically to both referral pathways.

Secondary outcomes reflected pathological severity at diagnosis and included Breslow thickness, mitotic rate, ulceration, and grouped AJCC 8th edition stage at excision. Stage 0 and stage I tumors were combined as an early-stage category, and lesions with Breslow thickness greater than 2.0 mm were analyzed as a marker of higher-risk disease burden. Pathology reports were reviewed from institutional records, and staging was assigned using AJCC 8th edition criteria based on Breslow thickness, ulceration, nodal status when available, and documented pathologic stage. Sentinel lymph node biopsy was recorded as a management indicator. Decisions regarding sentinel lymph node biopsy were made according to routine melanoma practice, considering Breslow thickness, ulceration, mitotic activity, patient comorbidity, and multidisciplinary judgment; therefore, SLNB rates were interpreted cautiously and not used as a direct surrogate for pathway severity.

### 2.4. Statistical Analysis

**Figure 1:**
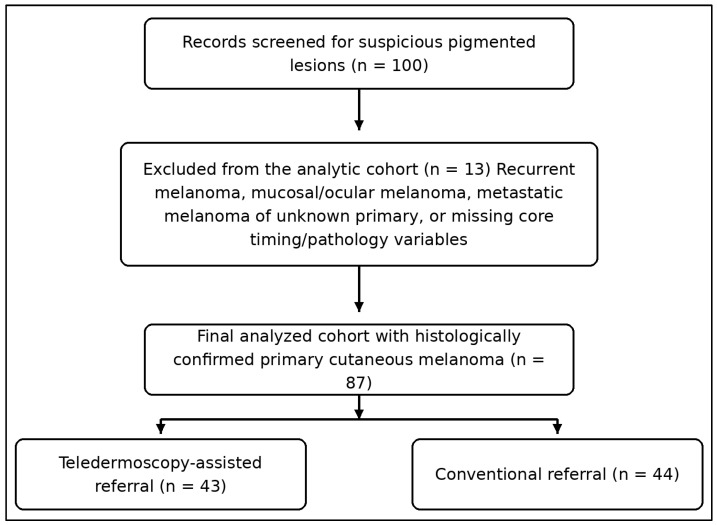
Participant flow diagram.

Age was treated as approximately normally distributed and is reported as mean ± standard deviation, with between-group comparison by Welch’s *t*-test. Time intervals, Breslow thickness, and mitotic rate were treated as non-normally distributed and are reported as median with interquartile range; these variables were compared using the Mann–Whitney U test. Categorical variables are presented as counts and percentages and were analyzed using Pearson’s chi-square test when multi-category comparison was appropriate or Fisher’s exact test when expected cell counts were small. All tests were two-sided, and *p* < 0.05 was considered statistically significant. To reduce reliance on *p*-values alone, absolute risk differences, risk ratios, odds ratios, and 95% confidence intervals were added for clinically important binary outcomes, including early-stage disease and Breslow thickness > 2.0 mm. A residence-stratified Cochran–Mantel–Haenszel odds ratio was calculated for early-stage disease as a limited sensitivity analysis. More extensive multivariable or propensity-score adjustment was not performed because the sample size was modest and several key allocation-related variables, such as lesion conspicuity, referrer suspicion, formal urgency category, patient delay before referral, and calendar-time service capacity, were not available for all patients. Spearman’s rank correlation coefficient was used only as an exploratory analysis of relationships between pathway intervals and pathological severity. These correlations were interpreted cautiously because between-pathway separation may contribute to the whole-cohort association (Figure 1).

## 3. Results

Baseline characteristics were generally similar between the two referral pathways for the measured demographic and referral variables; however, the groups should not be considered fully exchangeable because pathway assignment was not randomized and unmeasured clinical factors may have influenced referral type. Mean age was similar between the teledermoscopy-assisted and conventional groups (57.3 ± 13.6 vs. 60.3 ± 12.0 years, *p* = 0.278), as was the proportion of men (60.5% vs. 52.3%, *p* = 0.519). Rural residence was also evenly represented (44.2% vs. 52.3%, *p* = 0.522), which is important given the residence-based subgroup analysis. Most patients in both groups were referred from primary care, although the proportion was numerically higher in the teledermoscopy arm (83.7% vs. 70.5%, *p* = 0.203). Lesion distribution by body site did not show a significant overall difference (*p* = 0.470): trunk lesions were somewhat more frequent in the conventional pathway (47.7% vs. 32.6%), whereas head/neck lesions were somewhat more common in the teledermoscopy pathway (25.6% vs. 15.9%). Lower limb lesions accounted for 20.9% and 15.9% of cases, respectively, and upper limb lesions were almost identical between groups (20.9% vs. 20.5%), as described in Table 1.

The most pronounced between-group differences were observed in pathway timeliness, with teledermoscopy-assisted referral associated with shorter intervals across every measured milestone (Table 2). Median time from referral to dermatology consultation was reduced by approximately 9.7 days in the teledermoscopy group (9.0 [8.1–10.2] vs. 18.7 [16.3–20.7] days, *p* < 0.001). The advantage persisted through biopsy, which occurred 13.7 days earlier on median (16.6 [15.4–17.8] vs. 30.3 [28.9–32.0] days, *p* < 0.001), and definitive excision, which was completed 16.3 days earlier (26.9 [25.3–28.2] vs. 43.2 [41.2–46.6] days, *p* < 0.001). The pathway difference was not limited to front-end specialist triage. Teledermoscopy-assisted cases also moved more quickly from consultation to biopsy (7.6 [6.9–8.4] vs. 12.2 [11.2–13.1] days, *p* < 0.001) and from biopsy to excision (10.3 [9.2–11.6] vs. 13.5 [12.3–14.4] days, *p* < 0.001).

**Figure 2:**
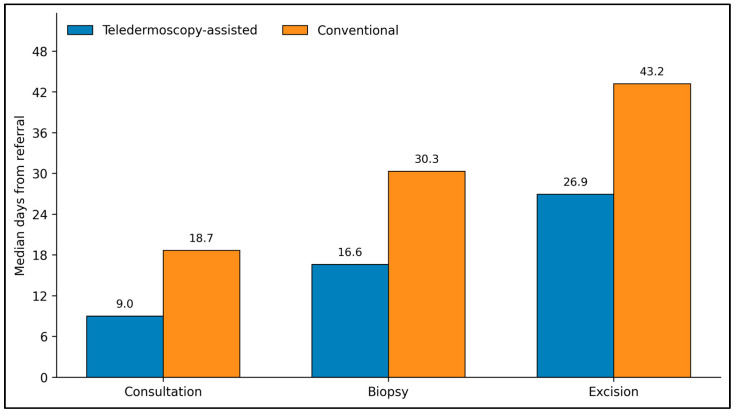
Median time from referral to dermatology consultation, biopsy, and definitive excision according to referral pathway.

Figure 2 summarizes the consistent reduction in time-to-event endpoints associated with teledermoscopy-assisted referral. The separation between groups is substantial across all three core milestones. Patients managed through teledermoscopy reached dermatology consultation at a median of 9.0 days compared with 18.7 days in the conventional pathway, showing that image-assisted triage was associated with a shorter time to specialist review. A similar pattern was evident for biopsy, where teledermoscopy reduced the median interval from 30.3 to 16.6 days, and for definitive excision, where the interval decreased from 43.2 to 26.9 days. This figure provides an overview of pathway compression across the melanoma workup rather than emphasizing only one step in isolation.

Histopathologic findings differed between pathways, but these differences should be interpreted as associations rather than proof that a 10-to-16-day acceleration alone caused stage migration. Median Breslow thickness was lower in the teledermoscopy group compared with the conventional pathway (0.7 [0.4–1.0] vs. 1.5 [0.8–2.4] mm, *p* < 0.001). Mitotic activity followed the same pattern, with a lower median mitotic rate in teledermoscopy-managed patients (1.2 [0.9–1.7] vs. 2.9 [1.6–3.8] mitoses/mm^2^, *p* < 0.001). Ulceration did not differ significantly (18.6% vs. 22.7%, *p* = 0.792). Stage 0/I melanoma was identified in 79.1% of teledermoscopy cases versus 40.9% of conventional referrals (*p* < 0.001), while stage III disease was less frequent in the teledermoscopy group (4.7% vs. 20.5%). Likewise, Breslow thickness > 2.0 mm was observed in 9.3% of teledermoscopy patients compared with 36.4% of conventional patients (*p* = 0.004), as seen in Table 3. Effect estimates and 95% confidence intervals for the main binary outcomes are provided in Table 4.

Additional effect estimates supported the clinical magnitude of the observed associations while emphasizing the uncertainty expected in a modest non-randomized cohort. Early-stage disease was more frequent in the teledermoscopy pathway, with an absolute risk difference of 38.2 percentage points, a risk ratio of 1.93, and an odds ratio of 5.46. Breslow thickness > 2.0 mm was less frequent in the teledermoscopy pathway, with an absolute risk difference of −27.1 percentage points, a risk ratio of 0.26, and an odds ratio of 0.18. A residence-stratified sensitivity analysis for early-stage disease yielded a similar association (Cochran–Mantel–Haenszel odds ratio 5.34, 95% CI 2.06–13.83), suggesting that the early-stage finding was not explained solely by urban/rural case mix (Table 4).

**Figure 3:**
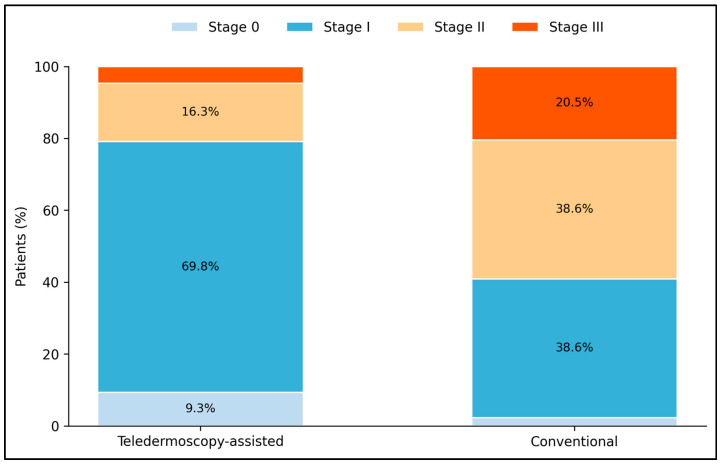
Stage distribution at diagnosis in the teledermoscopy-assisted and conventional referral pathways.

Figure 3 highlights the stage distribution observed in each pathway. The teledermoscopy pathway was dominated by stage I melanoma, which accounted for 69.8% of cases, and stage 0 lesions added another 9.3%, yielding a combined early-stage proportion of 79.1%. In contrast, the conventional pathway showed a more even distribution between stage I and stage II disease (both 38.6%), a lower proportion of stage 0 melanoma (2.3%), and a higher proportion of stage III cases (20.5% vs. 4.7%). Because pathway assignment was not randomized, this figure should be read as showing an observed stage profile difference rather than demonstrating that the shorter pathway interval alone caused the difference.

Operational quality indicators showed large differences between pathways (Table 5). Every patient in the teledermoscopy group was seen by dermatology within 14 days (43/43, 100.0%), whereas only 2 of 44 conventional referrals (4.5%) met that benchmark (*p* < 0.001). Biopsy within 18 days was achieved in 79.1% of teledermoscopy-assisted cases but in none of the conventional referrals (34/43 vs. 0/44, *p* < 0.001). Definitive excision within 40 days was likewise universal in the teledermoscopy pathway (100.0%) but uncommon in the conventional group (9.1%, *p* < 0.001). Selected downstream management indicators were similar between groups: sentinel lymph node biopsy was performed in 58.1% of teledermoscopy cases and 61.4% of conventional cases (*p* = 0.829), while positive sentinel nodes among biopsied patients were nearly identical (12.0% vs. 11.1%, *p* = 1.000). These SLNB findings should be interpreted in light of guideline-based eligibility, patient-level factors, and small event counts rather than as evidence that nodal-risk profiles were identical.

Residence-based subgroup analysis showed that the teledermoscopy-associated timeliness advantage was present in both urban and rural populations, with particularly relevant absolute differences among rural patients (Table 6). In urban residents, teledermoscopy shortened median time to consultation from 18.9 to 8.5 days and reduced referral-to-excision time from 42.4 to 26.5 days, both *p* < 0.001. Urban patients also had thinner melanomas in the teledermoscopy pathway (0.7 vs. 1.3 mm, *p* = 0.007) and a higher early-stage rate (83.3% vs. 42.9%, *p* = 0.006). Among rural patients, median time to consultation was reduced from 18.6 to 9.8 days (*p* < 0.001), and median time to excision fell from 44.7 to 27.2 days (*p* < 0.001). Early-stage melanoma was present in 73.7% of rural teledermoscopy cases compared with 39.1% of rural conventional cases (*p* = 0.033). These subgroup results are hypothesis-generating because the sample size was limited.

Correlation analysis demonstrated positive whole-cohort associations between longer pathway intervals and greater pathological severity; however, these findings were treated as exploratory because the separation between referral pathways may contribute to the observed correlations (Table 7). For referral-to-consultation delay, correlations were positive with Breslow thickness (rho = 0.495), mitotic rate (rho = 0.592), and stage (rho = 0.522). Similar results were observed for referral-to-biopsy delay, which correlated with Breslow thickness (rho = 0.462), mitotic rate (rho = 0.542), and stage (rho = 0.489). Referral-to-excision delay also correlated with Breslow thickness (rho = 0.540), mitotic rate (rho = 0.592), and stage (rho = 0.535). These results support the descriptive pattern that longer intervals and more severe pathology co-occurred in this dataset, but they should not be interpreted as proving a within-pathway biological gradient.

**Figure 4:**
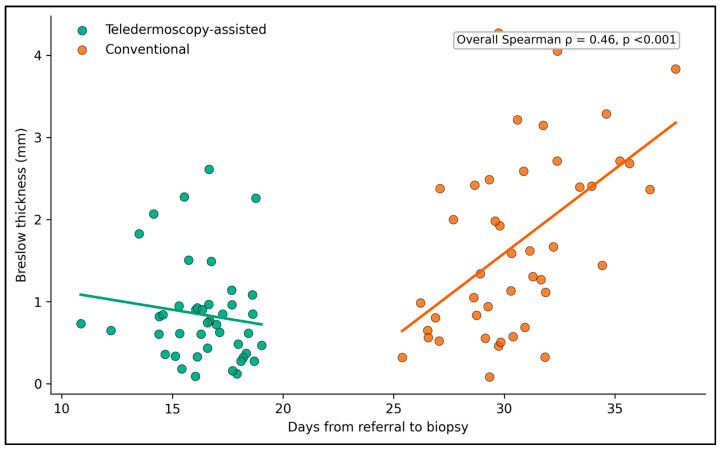
Relationship between time from referral to biopsy and Breslow thickness, stratified by pathway. Lines represent simple linear trend estimates for visualization.

Figure 4 illustrates the relationship between referral-to-biopsy delay and Breslow thickness, stratified by referral pathway. Teledermoscopy-assisted cases cluster in the lower-left portion of the figure, reflecting shorter biopsy intervals and thinner tumors, consistent with the group medians of 16.6 days and 0.7 mm. Conventional referrals shift toward longer time to biopsy and greater tumor thickness, with medians of 30.3 days and 1.5 mm. The fitted trend lines are intended for visualization only; because pathway membership itself separates the clusters, the figure should be interpreted descriptively rather than as definitive evidence of a causal delay–thickness relationship.

## 4. Discussion

### 4.1. Analysis of Findings

This comparative cohort suggests that teledermoscopy-assisted referral was associated with a shorter melanoma diagnostic pathway from its earliest stages. Compared with the conventional process, the image-assisted pathway showed shorter time to dermatologist review, biopsy, and definitive excision. The consistency of that advantage across the major intervals is important because it implies a pathway-level service effect rather than an isolated gain at the first appointment. This interpretation is broadly aligned with earlier teledermatology and teledermoscopy evaluations showing faster specialist decision-making, shorter triage intervals, and clinically useful agreement when image-enabled pathways are embedded in structured referral systems [16,17,18,19,20].

The pathological findings were clinically notable but require cautious interpretation. Patients in the teledermoscopy-assisted arm had thinner melanomas, lower mitotic activity, and a higher proportion of stage 0/I disease. These observations are compatible with the hypothesis that image-assisted triage may improve the pathway through earlier specialist prioritization; however, the non-randomized design prevents attribution of the full stage difference to a median acceleration of approximately 10–16 days. It is plausible that lesion conspicuity, referrer suspicion, patient delay, referral urgency, or calendar-time changes contributed to pathway assignment and tumor severity [21,22,23,24]. Therefore, the present findings are best viewed as a strong pathway-level association and a signal for prospective evaluation rather than proof of causal stage migration [25,26,27].

The residence-based subgroup analysis adds a service-planning dimension to the findings. Teledermoscopy-assisted referral improved timing in both urban and rural patients, and the rural subgroup showed numerically meaningful differences in Breslow thickness and early-stage disease. This pattern is directionally consistent with the broader telemedicine literature, in which smartphone-enabled image pathways, cost-conscious teledermoscopy models, and service reconfiguration strategies are viewed as tools that may reduce travel-related barriers, improve prioritization, and preserve access when traditional care pathways are strained [28,29,30]. Nevertheless, residence-stratified results should be considered hypothesis-generating because subgroup sizes were modest. Nevertheless, these findings should be interpreted in light of potential residual confounding from unmeasured or incompletely controlled factors, including underlying comorbidities and other patient- and treatment-related characteristics [31,32,33,34,35,36].

### 4.2. Study Limitations

This study has several limitations that should be considered when interpreting the findings. First, the observational single-center design limits causal inference and may reduce generalizability to other health systems with different referral structures, access constraints, or dermatology capacity. Second, the sample size was modest, particularly for subgroup analyses and less frequent outcomes such as sentinel node positivity, which limited the ability to detect smaller between-group differences and constrained multivariable adjustment. Third, although measured demographic variables were broadly similar, pathway allocation was not randomized and may have been systematically influenced by lesion conspicuity, referrer suspicion, formal urgency category, patient help-seeking behavior, pre-referral delay, and calendar-time service capacity. These unmeasured factors could influence both pathway assignment and tumor severity, meaning that residual confounding and selection bias are likely important rather than minor considerations. Fourth, the magnitude of the observed histopathologic and stage differences is larger than would be expected from the measured pathway delay alone; therefore, the results should not be interpreted as showing that a 10-to-16-day difference independently caused the entire Breslow or stage difference. Fifth, the analysis focused on diagnostic timeliness and stage-related measures at excision, without long-term oncologic outcomes such as recurrence, disease-specific survival, or overall survival. Finally, workflow-specific factors such as image quality, referrer expertise, pathology turnaround, and local scheduling logistics were not modeled separately, so the observed benefit should be interpreted as a pathway-level association rather than as the isolated effect of teledermoscopy itself.

## 5. Conclusions

In this non-randomized comparative cohort, teledermoscopy-assisted referral was associated with a substantially faster melanoma diagnostic pathway and a more favorable histopathologic profile at excision. Compared with conventional referral, the teledermoscopy-assisted pathway shortened time to consultation, biopsy, and definitive excision and increased attainment of predefined service benchmarks. The pathway was also associated with thinner melanomas, lower mitotic activity, and a higher proportion of stage 0/I disease, but these pathological differences should be interpreted cautiously because unmeasured referral and lesion-level factors may have contributed to pathway assignment. Taken together, the findings support teledermoscopy-assisted referral as a promising pathway-level service intervention for improving melanoma triage efficiency, while highlighting the need for prospective, adequately adjusted studies to determine whether such pathways independently improve stage at diagnosis or long-term patient outcomes.

## Figures and Tables

**Table 1:** Baseline demographic and referral characteristics by pathway.

Variable	Teledermoscopy-Assisted (*n* = 43)	Conventional (*n* = 44)	*p*-Value	Test
Age, years	57.3 ± 13.6	60.3 ± 12.0	0.278	Welch’s *t*-test
Male sex	26 (60.5)	23 (52.3)	0.519	Fisher’s exact
Rural residence	19 (44.2)	23 (52.3)	0.522	Fisher’s exact
Primary care referral	36 (83.7)	31 (70.5)	0.203	Fisher’s exact
Trunk lesion	14 (32.6)	21 (47.7)	0.470	Chi-square
Lower limb lesion	9 (20.9)	7 (15.9)		
Upper limb lesion	9 (20.9)	9 (20.5)		
Head/neck lesion	11 (25.6)	7 (15.9)		

Values are reported as mean ± standard deviation or *n* (%). Overall lesion-site distribution was compared using the chi-square test.

**Table 2:** Pathway timeliness outcomes.

Outcome, Days	Teledermoscopy-Assisted (*n* = 43)	Conventional (*n* = 44)	*p*-Value	Test
Referral to dermatology consultation	9.0 [8.1–10.2]	18.7 [16.3–20.7]	<0.001	Mann–Whitney U
Referral to biopsy	16.6 [15.4–17.8]	30.3 [28.9–32.0]	<0.001	Mann–Whitney U
Referral to excision	26.9 [25.3–28.2]	43.2 [41.2–46.6]	<0.001	Mann–Whitney U
Consultation to biopsy	7.6 [6.9–8.4]	12.2 [11.2–13.1]	<0.001	Mann–Whitney U
Biopsy to excision	10.3 [9.2–11.6]	13.5 [12.3–14.4]	<0.001	Mann–Whitney U

Values are reported as median [interquartile range]. Continuous between-group comparisons used the Mann–Whitney U test.

**Table 3:** Histopathologic severity and stage at diagnosis.

Variable	Teledermoscopy-Assisted (*n* = 43)	Conventional (*n* = 44)	*p*-Value	Test
Breslow thickness, mm	0.7 [0.4–1.0]	1.5 [0.8–2.4]	<0.001	Mann–Whitney U
Mitotic rate, mitoses/mm^2^	1.2 [0.9–1.7]	2.9 [1.6–3.8]	<0.001	Mann–Whitney U
Ulceration present	8 (18.6)	10 (22.7)	0.792	Fisher’s exact
Stage 0	4 (9.3)	1 (2.3)	0.003	Chi-square
Stage I	30 (69.8)	17 (38.6)		
Stage II	7 (16.3)	17 (38.6)		
Stage III	2 (4.7)	9 (20.5)		
Early stage (0/I)	34 (79.1)	18 (40.9)	<0.001	Fisher’s exact
Breslow > 2.0 mm	4 (9.3)	16 (36.4)	0.004	Fisher’s exact

Continuous variables are shown as median [interquartile range]. Stage distribution was compared using the chi-square test.

**Table 4:** Effect estimates with 95% confidence intervals for clinically important binary outcomes.

Outcome	Absolute Risk Difference (95% CI)	Risk Ratio (95% CI)	Odds Ratio (95% CI)	Residence-Adjusted OR (95% CI)
Early-stage disease (stage 0/I)	38.2 percentage points (17.7 to 54.4)	1.93 (1.31 to 2.85)	5.46 (2.11 to 14.10)	5.34 (2.06 to 13.83)
Breslow thickness > 2.0 mm	−27.1 percentage points (−42.9 to −9.5)	0.26 (0.09 to 0.70)	0.18 (0.05 to 0.59)	

The residence-adjusted estimate for early-stage disease used a Cochran–Mantel–Haenszel odds ratio stratified by urban/rural residence. More extensive adjustment was not performed because patient-level urgency, lesion-conspicuity, and pre-referral-delay variables were unavailable.

**Table 5:** Pathway-quality indicators and selected management outcomes.

Indicator	Teledermoscopy-Assisted (*n* = 43)	Conventional (*n* = 44)	*p*-Value	Test
Dermatology consultation ≤ 14 days	43 (100.0)	2 (4.5)	<0.001	Fisher’s exact
Biopsy ≤ 18 days	34 (79.1)	0 (0.0)	<0.001	Fisher’s exact
Excision ≤ 40 days	43 (100.0)	4 (9.1)	<0.001	Fisher’s exact
Sentinel lymph node biopsy performed	25 (58.1)	27 (61.4)	0.829	Fisher’s exact
Positive sentinel node among biopsied patients	3 (12.0)	3 (11.1)	1.000	Fisher’s exact

All categorical comparisons used Fisher’s exact test. Percentages for positive sentinel nodes are calculated among biopsied patients.

**Table 6:** Residence-based subgroup analysis.

Subgroup	Teledermoscopy-Assisted	Conventional	*p*-Value	Test
Urban patients	*n* = 24	*n* = 21		
Referral to consultation, days	8.5 [7.4–9.4]	18.9 [15.3–19.6]	<0.001	Mann–Whitney U
Referral to excision, days	26.5 [25.0–27.8]	42.4 [41.1–44.1]	<0.001	Mann–Whitney U
Breslow thickness, mm	0.7 [0.4–0.9]	1.3 [0.6–2.0]	0.007	Mann–Whitney U
Early stage (0/I)	20 (83.3)	9 (42.9)	0.006	Fisher’s exact
Rural patients	*n* = 19	*n* = 23		
Referral to consultation, days	9.8 [8.7–10.3]	18.6 [16.4–20.8]	<0.001	Mann–Whitney U
Referral to excision, days	27.2 [25.7–28.5]	44.7 [41.4–47.4]	<0.001	Mann–Whitney U
Breslow thickness, mm	0.8 [0.4–1.2]	1.9 [0.9–2.7]	0.007	Mann–Whitney U
Early stage (0/I)	14 (73.7)	9 (39.1)	0.033	Fisher’s exact

Continuous variables are shown as median [interquartile range]. Early-stage disease refers to stage 0 or stage I melanoma.

**Table 7:** Correlations between pathway delay and pathological severity in the whole cohort.

Variable Pair	Spearman Rho	*p*-Value
Referral to consultation vs. Breslow thickness	0.495	<0.001
Referral to consultation vs. Mitotic rate	0.592	<0.001
Referral to consultation vs. Stage	0.522	<0.001
Referral to biopsy vs. Breslow thickness	0.462	<0.001
Referral to biopsy vs. Mitotic rate	0.542	<0.001
Referral to biopsy vs. Stage	0.489	<0.001
Referral to excision vs. Breslow thickness	0.540	<0.001
Referral to excision vs. Mitotic rate	0.592	<0.001
Referral to excision vs. Stage	0.535	<0.001

Spearman’s rank correlation coefficient was used for all analyses. Positive coefficients indicate greater pathological severity with longer delay.

## Data Availability

The data presented in this study are available on request from the corresponding author.
